# Associations do not energize behavior: on the forgotten legacy of Kurt Lewin

**DOI:** 10.1007/s00426-021-01631-1

**Published:** 2021-12-24

**Authors:** Andreas B. Eder, David Dignath

**Affiliations:** 1 Department of Psychology, JMU Würzburg, Röntgenring 10, 97070 Würzburg, Germany; 2 Department of Psychology, University of Tübingen, Schleichstraße 4, 72076 Tübingen, Germany

## Abstract

Hundred years ago, Kurt Lewin published a series of articles in which he vehemently argued against the idea that associations between stimuli and responses motivate behavior. This article reviews his empirical work and theory and the cogency of Lewin’s conclusion according to modern standards. We conclude that Lewin’s criticism of the contiguity principle of associationism is still valid, and is now supported by a broad range of theories on learning, motivation, and action control. Implications for modern dual-system theory and modern theories on motivated action and (instructed) task sets are discussed.

Can mental associations energize behavior? In the first issues of the journal *Psychologische Forschung* (now *Psychological Research*), psychologist Kurt Lewin published two articles entitled “The problem of the measurement of will and the basic law of association. I and II,” in which he strongly argued against the view that mental associations energize behavior (Lewin, 1922a, 1922b). Hundred years after the publication, the questions discussed by Kurt Lewin are still timely. The notion that associations could activate behavior is contained in modern theories that claim an associative basis of “habitual”, “scripted”, or “impulsive” action tendencies (e.g., Abelson, 1981; Dickinson, 1985; Evans & Stanovich, 2013; Huesmann, 1988; Neal et al., 2006). The common underlying theme is that the repeated co-occurrence of a stimulus and a behavior (i.e., contiguity) creates an associative link between these elements, so that, *ceteris paribus*, activation of one element (the stimulus) will automatically activate the other element (the behavior). This contiguity principle of associationism states that the mere perception of a stimulus can be sufficient to energize the associated behavior via a spread of activation. However, as Lewin has argued, this view is not plausible and, as we will conclude in this review, his skeptical stance is now shared by many influential theories on learning, motivation, and action control.

## The life and work of Kurt Lewin in Berlin

Kurt Lewin was an assistant at the Institute of Psychology in Berlin at the time of the publication of his 1922 articles. In 1911, he started a doctorate study at the University of Berlin under the auspices of Carl Stumpf who was the director of the psychological laboratory and a dedicated ‘experimentalist’ (Perlina, 2015). Lewin, however, interrupted his study when he volunteered for military service in World War I during which he was also wounded. Nevertheless, he managed to receive his doctorate degree in 1916 and his ‘Habilitation’ (the formal qualification for a university lecturer position in Germany) in 1921. During his stay in Berlin, he was in close contact with the main protagonists of the Berlin Gestalt psychology—Max Wertheimer, Wolfgang Köhler, and Kurt Koffka—who founded the journal *Psychologische Forschung* together with Kurt Goldstein and Hans Walter Gruhle in the year 1921. This close relationship likely explains why *Psychologische Forschung* became his preferred publication outlet until his emigration to USA in the year 1933.

In his publications “The problem of the measurement of will and the basic law of association. I and II,” which appeared in the inaugural issues 1 and 2 of the journal, Lewin reported experiments that he conducted in Berlin between 1911 and 1914 for his Ph.D. and Habilitation projects. In total, he performed 31 studies (“Versuchsreihen”) of which 12 were included in the report and a preliminary report (“vorläufige Mitteilung”) which appeared in the journal *Zeitschrift für Psychologie* a few years earlier (Lewin, 1917, 1922a, 1922b). In the following, we refer to these three publications when we write of “Lewin’s articles” or “Lewin’s publications”. The main topic of his research was the experimental study of “activities of the will” using a quantitative measurement technique first established by Ach (1905). Lewin’s publications did not receive much attention from contemporary research fellows and by subsequent generations. In fact, a citation analysis (performed with Google Scholar at 11.8.2021) yielded 87 citations for Part I and 52 citations for Part II—which is relatively low in comparison to impressive 760 citations for Lewin’s principal work “Intention, Will, and Need” (“Vorsatz, Wille und Bedürfnis”) that was published in the same journal a few years later (Lewin, 1926). A major cause of the low international reception certainly was the language barrier for the then-dominating English reading scientific community after World War II (in contrast to his late work, his early articles were not translated into English). The articles are also massive reads with more than 200 journal pages and many may have regarded them as highly overlapping with his subsequent work (especially with Lewin, 1926). Furthermore, the bulk of the reported experiments yielded null effects, and the experimental methods, which were top-notch at his time, could be criticized as measured by modern standards (e.g., single-participant research designs; no inferential statistics). Although Lewin subsequently became one of the most famous German psychologists, his early studies are now known to only few contemporary scientists, which in our opinion is unfortunate given the topical discussions they contain. The aim of this article is to change this.

## Ach’s measurement of will by associative equivalence

Lewin’s studies were based on the pioneering work of the German psychologist Narziß Ach who introduced a quantitative measurement technique of “activities of the will” in the early years of the twentieth century (Ach, 1905, 1910, 1935). Ach’s combined method was straightforward: In a first (learning) stage, habitual action tendencies were established by overlearning particular responses to specific stimuli. In Ach’s studies, for example, the participants were asked to repeatedly read and learn lists and pairs of meaningless syllables (e.g., *ron lim fäb nuk*) that were presented on a mechanized memory drum. In line with the memory research of his mentor Georg Elias Müller (Müller & Schumann, 1894), Ach hypothesized that, after many learning trials distributed across several training days, the next syllable in the list would automatically pop up in the participant’s mind on the presentation of a syllable, generating a persisting tendency to reproduce the next syllable. This “reproductive tendency” must then be overcome in the second (test) stage of the experiment, for which the participant was asked to perform a different (so-called *heterogeneous*) activity on each syllable. For example, instructions of heterogeneous activities were to generate a rhyming syllable (rhyming task; e.g., *ron-nöm*) or to rearrange the letters (rearrangement task; e.g., *ron-nor*). Ach assumed that the participant must expend willpower to overcome the previously established reproductive tendency, which should cost time and/or result in systematic errors. This was indeed observed in many experiments (for a summary, see Ach, 1935).

Ach (1910, 1935) theorized that behavioral performance during the test phase is determined by a dynamic competition between the association-driven “reproductive response tendency” (the habit), that was established in the learning phase, and the intention-driven “deterministic response tendency” (the will), that was established for the test phase with the task instruction of the heterogeneous activity. In line with the contiguity principle of associationism, association formation, and with it the reproductive tendency, could be experimentally strengthened by increasing the number of learning trials; in contrast, the capacity for willpower is largely determined by personal factors such as the temperament of the person. By experimentally manipulating the number of practice trials, underlying associative strength, one can hence measure willpower quantitatively, because, theoretically, the person must expend more willpower to overcome a reproductive tendency after, let us say, hundred learning trials compared to only 50 learning trials. Ach speaks of an “associative equivalence” if the associative strength (manipulated via the number of learning trials) is on a par with the person’s willpower, as expressed by occasional but systematic errors to reproduce the previously learned syllable in the test phase.

## The studies of Kurt Lewin

Kurt Lewin started his research with an investigation of how the list length would affect the strength of reproductive–deterministic tendencies using Ach’s combined paradigm. His technical equipment was very modern according to the standards of his time, consisting of an automated memory drum and the registration of vocal reactions in the range of a few seconds using a Hipp chronoscope that was wired to a self-constructed voice trigger (Lewin, 1922c). His research interest, however, shifted when his experiments repeatedly failed to demonstrate interference effects after extensive syllable learning (with a maximum of 300 learning trials for each syllable). As a result, his discussion centered on the “psychology of associations” and, in particular, the contiguity principle which he referred to as a fundamental law of associationism. He described this law in the following way: “If two entities have frequently entered consciousness (at the same time or in immediate succession) and one of them becomes conscious again, then the other also has the tendency to reappear.”^1^ (Lewin, 1922a, p. 261). Lewin was very clear that his studies cannot test associationism per se but only the contiguity law as instantiated in Ach’s combined method.

In Part I, Lewin (1922a) reported nine studies that primarily differed in the numbers and type of learning trials and in the type of activities performed for the test phase. In a first set of studies (A-C), extensive practice of syllable learning did not interfere with performance (reaction times, errors) of a heterogeneous activity on that syllable (relative to syllables that were not presented in a particular order). It should be noted that Lewin’s procedure was not an exact replication of Ach’s original procedure, which became a topic for subsequent discussion (see, e.g., Simoneit, 1926). For example, Lewin presented different syllables with varying list length (2–16 syllables), whereas Ach presented only pairs of syllables that were constructed in a systematic way (e.g., rhyming pairs: *dus sud*). Furthermore, for comparisons, Ach included separate test blocks with a free reproduction task in which the participant could name any syllable, whereas Lewin alternately presented practiced and new (weakly practiced) syllables during the test phase, using a procedure introduced by one of Ach’s students (Glässner, 1912).

Lewin, however, could demonstrate with various controls and manipulation checks that participants have memorized the syllables: They read and recited the lists more than 200 times, extended over a time period of more than 2 weeks, and measurements at the end of the practice phase confirmed that the participant could recite the syllables quickly and without errors. Participants could also recollect the next syllable in a list without difficulty if explicitly asked to do so. These controls make it implausible that the practice phase was generally inappropriate for memorization in line with the contiguity principle.

Lewin consequently concluded that the frequent reading and recitation of syllable lists during the practice phase was not sufficient for the instigation and/or operation of a reproductive tendency in the test phase. In addition, he noted, based on an analysis of participants’ self-observations during the tasks, that they tended to identify the presented syllables into known/unknown items, which was an uninstructed activity that presumably delayed the execution of the instructed task during the test phase. Lewin theorized that the delay was stronger for the practiced syllables due to the increased complexity of the operation, accounting for a slowing down of the response which he occasionally observed in his studies.

Lewin’s research focus consequently shifted to a systematic investigation of the activities performed in each phase, and whether practice of a particular task would affect performance in the test phase differently depending on whether he or she had practiced the same (a *homogenous*), a different (a *heterogeneous*), or a neutral (*indifferent*) task with that syllable. In one study, for example, the participant practiced letter rearrangement and rhyming with separate lists of syllables, intermixed with so-called *variable* syllables that appeared in both lists with equal frequency. After extensive task practice, the syllables were presented again during the test phase but this time with instructions of the same task or a different task. Lewin hypothesized that, if a reproductive tendency was established by the repetition of a particular task-related activity during the practice phase, then rhyming of previously rhymed syllables should be faster, and rhyming of previously rearranged syllables should be slower in the test phase, compared to rhyming of variable syllables. Results, however, showed no performance differences depending on whether the syllable was practiced before with the same or different tasks. In a summary of this set of studies, he therefore concluded:

“This [the absence of a reproductive tendency] seems to prove that the law of association is not tenable in its current version, since the effects predicted by this law can be absent without any occluding factors, even if the conditions demanded by the law were fulfilled.*”*^2^ (Lewin, 1922a, p. 301).

In Part II, he examined conditions that must be met for the generation of a reproductive tendency (Lewin, 1922b). Specifically, he theorized that the participant must have a specific intention, or “action readiness” (“Tätigkeitsbereitschaft”) as he named it, to reproduce the next syllable in a list. He reasoned that the reproductive tendency was prevented in his previous studies by the intermixing of novel (the *variable*) syllables that required from the participant the construction of a new (rhyming) syllable on each syllable presentation. Consequently, he eliminated these syllables from the design and presented only lists of syllables that the participant must either rhyme or rearrange during the practice phase. In the subsequent test phase, participants continued with these tasks in separate blocks, but this time the list also contained a syllable that was previously used for the other task. Results showed that participants responded to these intermixed syllables very slowly, and sometimes even incorrectly with the previously practiced response. This interference effect was observed with a low number of only eight practice trials for each task. Lewin, therefore, concluded that the generation of a reproductive tendency does not depend on the frequency of contiguous presentations but, rather, on the participant’s readiness for reproduction, which is an intentional process.

Lewin’s research was in line with other criticism of Ach’s combined method that argued for a goal dependency of reproductive tendencies established with this procedure (Selz, 1910). Furthermore, one of Ach’s students (Simoneit, 1926) also found no interference after extensive syllable learning when using Lewin’s procedure with intermixed variable syllables; however, when Simoneit used Ach’s procedure without variable syllables, he still obtained an interference effect. Hence, a relatively minor procedural difference during the learning phase turned out to be critical for the observation of an interference effect, which was difficult to reconcile with Ach’s original assumption that the mere proximity of stimuli in space and time (contiguity) is sufficient for the generation of a reproductive tendency.

Ach (1935) therefore later admitted in his book, in a discussion of Lewin’s research, that the contiguity principle in its original formulation could not be valid and requires modification. However, he still adhered to the measurement approach with associative equivalence and that association-driven reproductive tendencies could be established with the correct procedure. Lewin, in contrast, rejected the idea that a mental connection between psychic elements (association), in itself, could be sufficient to energize a behavioral activity; for him, this energy comes from a person’s striving toward a particular action goal, which he theorized as a state of action readiness that operates independently from the contiguity principle.

## The door handle example

Lewin (1922b) illustrated his theoretical concept of “action readiness” using the everyday example of opening a door. During her lifetime, a person should have made countless experiences that a door can be opened with a pressing down the handle. According to the contiguity principle, these experiences should have left a memory trace, generating a strong habit to press down the door handle when in reach. In his thought experiment, Lewin imagined a door with a reversed mechanism that is opened by pushing the handle upwards. Would the person be able to open the door on the experimenter’s request if she knows that this door is opened by lifting the handle?

According to Ach’s account, an intense will act should be necessary to overcome the down-pressing habit established via countless repetitions. Lewin, by contrast, hypothesized that the person can execute the instructed action effortless on explicit request, because a new action readiness was formed on the experimenter’s instruction. Lewin’s thought experiment was not realized, but everyday intuition, and controlled laboratory studies using related setups, appear to fit more to Lewin’s prediction. For example, Wickens (1938) trained participants to avoid painful shocks signalled by a buzzer by pressing down a metal bolt with the middle finger. After sufficient training, the positioning of the arm was changed and the shock was now be avoided by lifting the finger. Results showed that most subjects executed immediately the reverse movement without difficulty—just as expected by Lewin for handling a reversed door opening mechanisms.

According to Lewin, however, the person should experience difficulties with opening the new door when her goal is to enter the next room. With this superordinate action goal, the downward push of the door handle is misapplied as a routine activity of entering the next room, even when the person has good knowledge of how this door can be opened. Action errors, such as opening the door in the practiced way, hence are only predicted for situations in which the routine activity is an integral part of another, superordinate goal-directed activity. Importantly, this integration process is triggered by the preparation of a superordinate activity or task—and hence under voluntary control.

Lewin discussed several ways how a readiness for action could develop without associative learning. First, a readiness to act could be formed by conscious, or even automated, acts of will. The conscious wilful act is typical for task settings in which participants must follow the instructions of the experimenter; however, it can also occur in the absence of explicit prompts when the person is free to decide on how to solve a problem or task. For example, Lewin speculated that Ach’s memorization instructions for the learning phase actually suggested a reproductive activity to the participant, although this was not explicitly mentioned in the task instruction. Furthermore, an action readiness could be formed by automated acts of will, for instance, when the activity is needed for a superordinate activity. In the door handle example described above, the downward push of the door handle is triggered automatically on the sight of the door handle, because door opening is an integral part of entering the next room. After preparation of the superordinate activity, the subroutine is automatically initiated without additional intervention by the conscious will.

Second, an existing action readiness could be transformed to another action readiness or it could be replaced by a better fitting one. Lewin mentioned the rhyming task as an example where the subject started with rhyming and, after some practice, switched to the reproduction of previously rhymed syllables. The readiness to produce a rhyming syllable was transformed to a readiness to reproduce previously generated (rhyming) syllables, presumably because the latter activity was more convenient for the participant.

Third, the action readiness could also be latent in the sense of a persisting disposition that was formed by inborn drives and/or educational practices. Lewin discussed as example the person’s readiness to identify an object as familiar, which exists independently of a conscious or automated decisional act. Lewin was aware of the vagueness of the concept, and in his article, he described several criteria for the identification of a latent action readiness (Lewin, 1922b, p. 99).

Action readiness includes a cognitive specification of the appropriate conditions on which the prepared action is released. Lewin referred to these conditions as “Aktivierungsreize” (“activating stimuli”). Examples from Lewin’s own studies are two-syllable words for which the instructed task was rhyming, but basically, every target of a planned response can become an activating stimulus. The concept of activating stimuli resembles Ach’s concept of a “referential idea” (“Bezugsvorstellung”) that specifies the necessary means for reaching a particular goal (for a modern interpretation of Ach’s original hypothesis, see the research on implementation intentions; Gollwitzer, 1999). Lewin, however, saw them as different, because he conceived activating stimuli as dynamic and, in principle, separable from conscious acts of will. For example, the apple becomes an activating stimulus for eating in a state of hunger but not in a state of satiety. The function of the apple as an activating stimulus consequently depends on a fluctuating inner need state (a latent action readiness in Lewin’s framework). However, the readiness to eat the apple could be also installed via other means, for example, by a mother’s explicit command to a child to eat healthy food (explicit act of will) or by virtue of being a side dish in a large meal (automated act of will). To summarize, according to Lewin, a readiness for action is established via relations to needs and quasi-needs (intentions), and the function of selected stimuli as “prompts for action” is dynamic, because it changes with transformation of the underlying action readiness.

The action readiness, once formed, will persist until the goal of the activity is reached or until it is replaced by another action readiness. Lewin explicitly included the stopping of an ongoing activity (“das Aufhören”) as a type of action readiness that could be wilfully instantiated. Furthermore, the instruction of a new task alone is often not sufficient for a change in action readiness, because the existing one must be actively removed or terminated. Lewin did not clarify how this is implemented on the process level; however, the theorizing fits to modern research on task set inertia (e.g., Ruthruff et al., 2001) and the demonstration of a persisting inhibition of abandoned task sets (for a review, see Koch et al., 2010).

## Learning, practice, and habit formation

The contiguity principle, as critically discussed by Kurt Lewin in his publications, was originally introduced as a basic principle of learning and memory recall by the German “associationists” Hermann Ebbinghaus and Georg Elias Müller. Ach justified the measurement idea of an associative equivalence with direct references to this pioneering research; and even though the numbers of learning trials turned out to be unimportant in his own studies, Lewin did not question the importance of practice and acquisition of behavioral skills. In his articles, he therefore discussed at length the development of habits and of skilled performance in general.

For this discussion, he distinguished so-called *need- or drive-based habits* (“Bedürfnis-/ Trieb-Gewohnheit”), that are directed at the satisfaction of a particular need (e.g., cocaine intake), from so-called *habits of execution* (“Ausführungsgewohnheit”). Both types often go hand in hand (for example, when the cocaine addict sniffs a surrogate powder, because the drug is not available), but only the habit of execution produces a practice effect in the sense that the behavior is executed faster and/or more readily with repetition.^3^ In addition, Lewin distinguished learning, which should result in association formation according to associationism, from practice of a learned behavior, which consolidates or strengthens the newly established associations. Lewin proposed an alternative account of learning and practice effects. In line with principles of Gestalt psychology (Koffka, 1935), learning consists for him in the *organization* of activities which involves a search and combination of activities that are appropriate for bringing success. Practice effects are produced by increasing the efficiency of action planning processes, which consists in (i) the elimination of detours, such as removing redundant movements and planning steps; and (ii) improvements in the coordination and timing of movements. This was a remarkable modern analysis of skill acquisition that is now supported by many research findings (Ericsson & Lehmann, 1996). For example, Gentner (1983) noted two general changes during acquisition of the typewriting skill: (1) the finger movements became less sequential and more overlapping with practice; and (2) performance shifted from being limited by cognitive constraints in students, to being limited by motoric and physical constraints in experts. The similarity to Lewin’s analysis is obvious.

According to Lewin, the skilled cognitive (re)organization of activities results in the formation of a cognitive action complex (“Tätigkeitskomplex” or “Komplextätigkeit”) that can be activated with a “single impulse of will”, which releases the behavior sequence in an automated fashion on a subconscious level. Again, one can see here a precursor of a modern action control perspective that posits the execution of a cognitively compiled “action plan” with minimal conscious intervention (Hommel, 2013; Kunde et al., 2012).

In addition, Lewin posited a distinction between knowledge updating and the acquisition of an action complex. Knowledge of an action must not be confused with the competency to perform an action, although he also emphasized the dynamic interaction between both during the early stages of action learning. New knowledge generates a subjective image (“subjektives Bild”) that can psychologically exist without generating a (reproductive) action tendency. Although Lewin did not fully elaborate the concept of a subjective image, it has much similarity to Tolman’s concept of a “cognitive map” and latent learning. Tolman (1932) gave Lewin’s work much coverage in his seminal book “On purposive behavior in animals and men”, and he was his close friend (he held his funeral eulogy); hence, it is likely that Tolman’s work was heavily influenced by Lewin’s theorizing on differences between knowledge and action structures.^4^

## Reception by modern psychology

As we have described above, Kurt Lewin argued in his articles vehemently against the idea that associations between stimuli and responses can energize behavior. A few years later, he summarized this position in the following way:

“The experimental investigation of habits (association) has shown that the couplings created by habit are never, as such, the motor of a psychical event. […]. Rather, certain psychical energies, that is, tense psychical systems which derive, as a rule, from the pressure of will or of a need, are always the necessary condition of the occurrence—in whatever way—of the psychical event. […]. For *connections are never causes of events* [emphasis in the original], wherever and in whatever form they may occur.” (Lewin, 1926; cited from an excerpt translated by D. K. Adams and K. E. Zener, 1935, pp. 44–45).

This conclusion did, however, not had a lasting impact on subsequent psychological theorizing, presumably because Lewin’s articles were subsequently mainly discussed as a major blow against Ach’s combined method and the German experimental will psychology (see, e.g., Heckhausen, 1987; Kuhl & Beckmann, 1985). It should be also noted Lewin did not include studies of classical (Pavlovian) and instrumental (Thorndikian) conditioning in his analyses, which are the poster child of associative learning (Hall, 2002; but see also Mitchell et al., 2009). The contiguity principle was also perpetuated by early neuroscientific theories, most notably the Hebbian learning model (“Neurons that fire together wire together”), which many viewed, and still continue to view, as the biological correlate of associative learning (for a critical discussion of this view, see, e.g., Gallistel & Matzel, 2013; McClelland, 2006). It is hence not surprising that the contiguity principle of associationism is well and alive in modern psychology and popularized in diverse research fields ranging from habit research (e.g., Neal et al., 2006), modern ideomotor theory (e.g., Elsner & Hommel, 2004), social cognitive script theory (e.g., Huesmann, 1988), to attitude research (e.g., Fazio, 1995; Greenwald et al., 1998), and dual-routes models (e.g., Evans & Stanovich, 2013). Ironically, it was the poster child (conditioning research) that experienced the greatest revision after modern accounts highlighted the importance of saliency (attention), informational pick-up from base rates (contingency), and rates of event occurrence (time-scale invariance) as mediating factors of conditioning (Baum, 1973; Gallistel & Gibbon, 2000; Pearce & Hall, 1980; Rescorla & Wagner, 1972). In addition, empirical studies demonstrated that contiguity is neither necessary (e.g., Garcia & Koelling, 1966) nor sufficient (blocking; Kamin, 1967) for association formation aka conditioning. This modern research confirms Lewin’s conclusion that the contiguity principle in its original formulation was too simplistic for a sufficient account of human learning.

Another reception of Lewin’s work analyzed the conditionality of highly overlearned “automatic” reactions and the contribution of action planning to this process. Hommel (2000) reviewed many research findings that demonstrated the dependence of automatic response tendencies in S–R compatibility tasks (e.g., spatial Simon and color Stroop tasks) on particular preparatory states. In line with Lewin’s position, he argued that intentional preparation sets the stage for automatic stimulus–response translation to task place. Research findings in line with this view accrued in the last 2 decades, for example, showing that task instructions alone, without practice of a response, can trigger automatic reactions (e.g., Cohen-Kdoshay & Meiran, 2009; Everaert et al., 2014; Meiran et al., 2015; for a review, see Meiran et al., 2017). Liefooghe et al. (2012) showed that automatic activation of instructed responses is reduced to nonsignificant levels when participants were required to memorize the instructions for later recognition instead of actual execution, and another study demonstrated that the instruction-based response activation depended on the degree of preparation for the upcoming task (Liefooghe et al., 2013). Although the exact nature of the preparatory state is still researched (e.g., Liefooghe & De Houwer, 2018; Monsell & Graham, 2021; Whitehead & Egner, 2018), the similarity to Lewin’s conjecture of an “action readiness” as a necessary condition for the generation of a reproductive tendency is striking.

Lewin’s concept of a cognitively prepared action complex (“Tätigkeitskomplex”), which can interfere with another action complex, also overlaps with the modern conceptualization of a ‘task set’ and switching between different task sets. Logan and Gordon (2001) defined a task set as a set of parameters that program task-specific processes such as perceptual encoding, memory retrieval, response selection, and response execution. This definition fits to Lewin’s notion of a ‘complex activity’ that consists of a coordinated set of subprocesses in the pursuit of a task goal. A task set includes the representation of task-relevant stimuli (or “activating stimuli” in Lewin’s terminology) and their relation to task-relevant responses in corresponding S–R mappings (the “referential idea” in Ach’s language) and could hence be viewed as a task-specific action readiness. Many studies demonstrated that switching between different tasks sets is costly (i.e., slow and error prone), and that the (re)configuration of a task set is governed by the task goal (endogenous control) and by task-relevant stimuli and their context (exogenous influences) (for reviews, see Monsell, 2003; Kiesel et al. 2010). In addition, the mere maintenance of alternative task sets in memory for task management takes a toll on performance (so-called *mixing costs*; Los, 1996; Rubin & Meiran, 2005). Although Lewin’s studies had no noticeable impact on the development of this modern research, the overlap of his account with modern accounts of task sets and task set reconfiguration is substantial.

Lewin’s early studies could be also read as important steps toward the development of his ‘topological approach’ that had a lasting influence on motivation and social psychology (Lewin, 1935, 1936, 1938). For this approach, “deterministic tendencies” (i.e., action intentions) are pervasive and ubiquitous psychological forces that influence how a person structures the perceived environment in respect to opportunities for action. For Lewin, *intended* actions do not qualitatively differ from other *motivated* actions, which is why he also referred to the former as “quasi-needs.” Multiple action tendencies could persist at any given time and could compete for realization in a situation, which adds a dynamic component to the model (for modern perspectives, see, e.g., Atkinson & Birch, 1970; Cisek, 2007). Curiously, this dynamic view is not so different from Ach’s conflict model between deterministic and reproductive action tendencies. In contrast to Ach, however, Lewin did not believe in associations as action energizers, because the source of action energy is always related to a person’s needs and wants.

This position was even acknowledged by early behavioristic theories that explained human behavior with conditioning and reinforcement principles. According to Hullian drive theory (Hull, 1943), organisms only behave for the satisfaction of a biological or physiological need, proposing that S–R habits must be fueled by the reduction of a biological drive to have a behavioral effect. Modern motivation theories abandoned the drive concept but retained energizing constructs such as motives, needs, and incentives (Berridge, 2001; Maslow, 1954; McClelland, 1987; Toates, 1986). The common underlying theme is that stimuli can acquire motivational properties by arousing matching needs and motives either by virtue of primitive associative links (Dickinson & Balleine, 2002) or via more complex cognitive operations involving value assignments and utility calculations (Eccles & Wigfield, 2002; Kahneman & Tversky, 1979). Either way, the connection itself is not a sufficient source of motivation according to these theories but, rather, the needs, values, and goals that are active in a situation.

Finally, one can relate Lewin’s research on a conflict between deterministic and reproductive response tendencies to modern theorizing about two distinct systems that compete for control of an overt response. For example, the Reflective-Impulsive Model (RIM; Strack & Deutsch, 2004) distinguishes (i) an impulsive system that activates behavioral schemata via processes of spreading activation; (ii) from a reflective system that activates behavior via rule-based decision processes. Importantly, the impulsive system is capable of activating a behavior without the person’s intention or goal, mediated by associative networks that directly link perceptual inputs to motor outputs (or more precisely, the cognitive representations thereof). The RIM assumes that “in general, [associative] links are created or strengthened if stimuli are presented or activated in close temporal or spatial proximity” (Strack & Deutsch, 2004, p. 223). Hence, the model endorsed the contiguity principle in a similar vein as the early associationists and Narziß Ach did hundred years ago in respect to the generation of a reproductive tendency. It is obvious that Lewin would have strongly opposed this dual-system view. First, he would have disagreed with the assumption that co-occurrence between stimuli and responses is sufficient for the generation of associative S–R clusters. Second, he would have objected to the notion that associations could energize behavior independently of a person’s goals and intentions. It is fitting that evidence cited by the RIM in favor of a direct (associative) perception-behavior link turned out to be not reproducible (see Chivers, 2019; Doyen et al., 2012; Sherman & Rivers, 2021) or was re-interpreted with motivational mechanisms (Cesario et al., 2006; Weingarten et al., 2016). It seems that history was repeating the Lewin-Ach controversy in a modern academic discourse and Lewin’s failures to find evidence for association-driven response tendencies.

The alternative to dual-system and dual-routes models are “unimodels” that explain competition between “will” and “habits” within a unitary approach (De Houwer, 2019; Hommel, 2019; Kruglanski, 2013; Melnikoff & Bargh, 2018). For example, Aarts and Dijksterhuis (2000) demonstrated in a series of studies that the automaticity of habitual behavior is conditional on the presence of an active goal, arguing that automatic “habitual” performance is actually the outcome of a formed implementation intention. Hommel and Wiers (2017) explained transitions between ‘intentional’ and ‘automatic’ action control modes with a singular mechanism that selects actions based on several weighted, and sometimes even conflicting, goal criteria. Kruglanski and Szumowska (2020) reviewed claims of “purposeless” habitual behavior in animal and human research and reached the conclusion that habitual performance in these studies was actually goal-driven. In line with Lewin’s early criticism of Ach’s dual-route model, modern research hence confirms that the “automatic” (reproductive) route is moderated and presumably even controlled by goals, while the “intentional” (deterministic) route often shows characteristics that are commonly associated with automaticity.

Is there any modern evidence for association formation according to the contiguity principle that could not be explained away with Lewin’s concept of action readiness? Frankly speaking, we believe that definite evidence cannot exist, because, according to Lewin, every learning and perceptual task must be guided by a motivation or instruction for the task. For Lewin, the associationists’ idealized situation that a contiguity between sensory events is passively registered by an organism is not real, because every perception is influenced by prior intentions and wants of the person for a situation.^5^ This means, Lewin’s alternative explanation is not falsifiable, because one can never convincingly rule out the existence, or generation, of (latent) preexisting needs and uninstructed quasi-needs that structure the person’s perception of the action task. Researchers can, however, investigate moderating effects of intentions and task instructions on the perception of contiguous events, which could demonstrate a relative independence from specific task-related intentions in line with the contiguity principle of associationism.

## Coda

Lewin’s early publications could, first and foremost, be read a methodological critique of Ach’s measurement idea of willpower with associative equivalence. It is, however, much more than this! In line with principles of Gestalt psychology (Koffka, 1935), they can be understood as a fundamental critique of associationist theories of human thought and behavior which claim that mental states could become associated by contiguity (i.e., by neighborhood in space and time), and that bringing one of a pair of associates to mind will, ceteris paribus, also activate the other (Mandelbaum, 2020). Their guiding principle is a *configuration* of elemental features and the spread of activation between these elements (“this goes with that”). Gestalt psychology, in contrast, emphasizes the *organization* of events and the interaction between these events (“the whole is other than the sum of its part”). For the Gestalt psychologist, proximity in space and time (contiguity) is just one organizing principle out of many (Köhler, 1941). As Lewin pointed out very clearly from the beginning, one cannot prove that Gestalt psychology is correct and associationism wrong (or vice versa), because, speaking in the language of Lakatos (1976), both have the status of a “scientific research program” that are not falsifiable. One can only derive testable theories from each program that are put to an empirical test. Lewin did so in respect to Ach’s idea of an associative equivalence and it did not pass the test, but this failure can be explained away with references to the “protective belt” of the theory (see Ach, 1935). Hundred years later, which has produced a myriad of new findings challenging the contiguity principle, it is the task for the proponents of associationism to demonstrate that the “hard core” assumption of action energization by association is tenable. Being a philosopher of science himself *and* a dedicated experimentalist, we believe that Lewin would have liked that.

## Footnotes

1 Original: “Sind zwei Gebilde häufig (gleichzeitig oder) unmittelbar hintereinander ins Bewußtsein getreten, und das eine von ihnen wird wieder bewußt, so hat auch das andere die Tendenz, wieder aufzutauchen.“

2 Original: „Damit scheint mir erwiesen, daß das Assoziationsgesetz in seiner bisherigen Fassung nicht haltbar ist, da die von diesem Gesetz geforderten Wirkungen, ohne daß verdeckende Faktoren vorliegen, ausbleiben können, auch wenn die vom Gesetz geforderten Bedingungen erfüllt sind.“

3 This difference is obvious in drug addiction when the action tendency (craving) for additional drug intake is temporarily reduced, and not enhanced, by consumption of the drug.

4 This was even explicitly noted by Tolman (1932/1967) in a footnote: “In general, it is to be said that a great deal of the doctrine to be outlined in the present treatise seems to bear a close relationship to the doctrine of Lewin, as the latter is to be gleaned from his writings and from those of his students.” (p. 37).

5 A related assumption is made by the “New Look” approach in psychology (Bruner & Goodman, 1947) and contemporary theories of motivated perception (Dunning & Balcetis, 2013; Proffitt, 2006).
